# Water oxidation in photosystem II

**DOI:** 10.1007/s11120-019-00648-3

**Published:** 2019-06-11

**Authors:** Wolfgang Lubitz, Maria Chrysina, Nicholas Cox

**Affiliations:** 1 Max-Planck-Institut für Chemische Energiekonversion, Mülheim/Ruhr, Germany; 2 Research School of Chemistry, The Australian National University, Canberra, Australia

**Keywords:** Photosystem II, Oxygen-evolving complex, Water binding, Triplet oxygen formation, EPR spectroscopy, Quantum chemical calculations

## Abstract

Biological water oxidation, performed by a single enzyme, photosystem II, is a central research topic not only in understanding the photosynthetic apparatus but also for the development of water splitting catalysts for technological applications. Great progress has been made in this endeavor following the report of a high-resolution X-ray crystallographic structure in 2011 resolving the cofactor site (Umena et al. in Nature 473:55–60, 2011), a tetra-manganese calcium complex. The electronic properties of the protein-bound water oxidizing Mn_4_O_x_Ca complex are crucial to understand its catalytic activity. These properties include: its redox state(s) which are tuned by the protein matrix, the distribution of the manganese valence and spin states and the complex interactions that exist between the four manganese ions. In this short review we describe how magnetic resonance techniques, particularly EPR, complemented by quantum chemical calculations, have played an important role in understanding the electronic structure of the cofactor. Together with isotope labeling, these techniques have also been instrumental in deciphering the binding of the two substrate water molecules to the cluster. These results are briefly described in the context of the history of biological water oxidation with special emphasis on recent work using time resolved X-ray diffraction with free electron lasers. It is shown that these data are instrumental for developing a model of the biological water oxidation cycle.

## Introduction

More than three billion years ago, the cyanobacteria evolved a light-driven enzyme that was able to split water into molecular oxygen and hydrogen. Biology couples this to the reduction of carbon dioxide (CO_2_) to carbohydrates and in so doing stores the energy of the sun in energy-rich compounds with water acting as the electron source. We call this whole process photosynthesis and it represents the central metabolic pathway of the biosphere. While there exists a wide variety of photosynthetic organisms from cyanobacteria to algae and higher plants, the cellular machinery responsible for water splitting is unique and uses the same mechanism across all species.

**Fig. 1:**
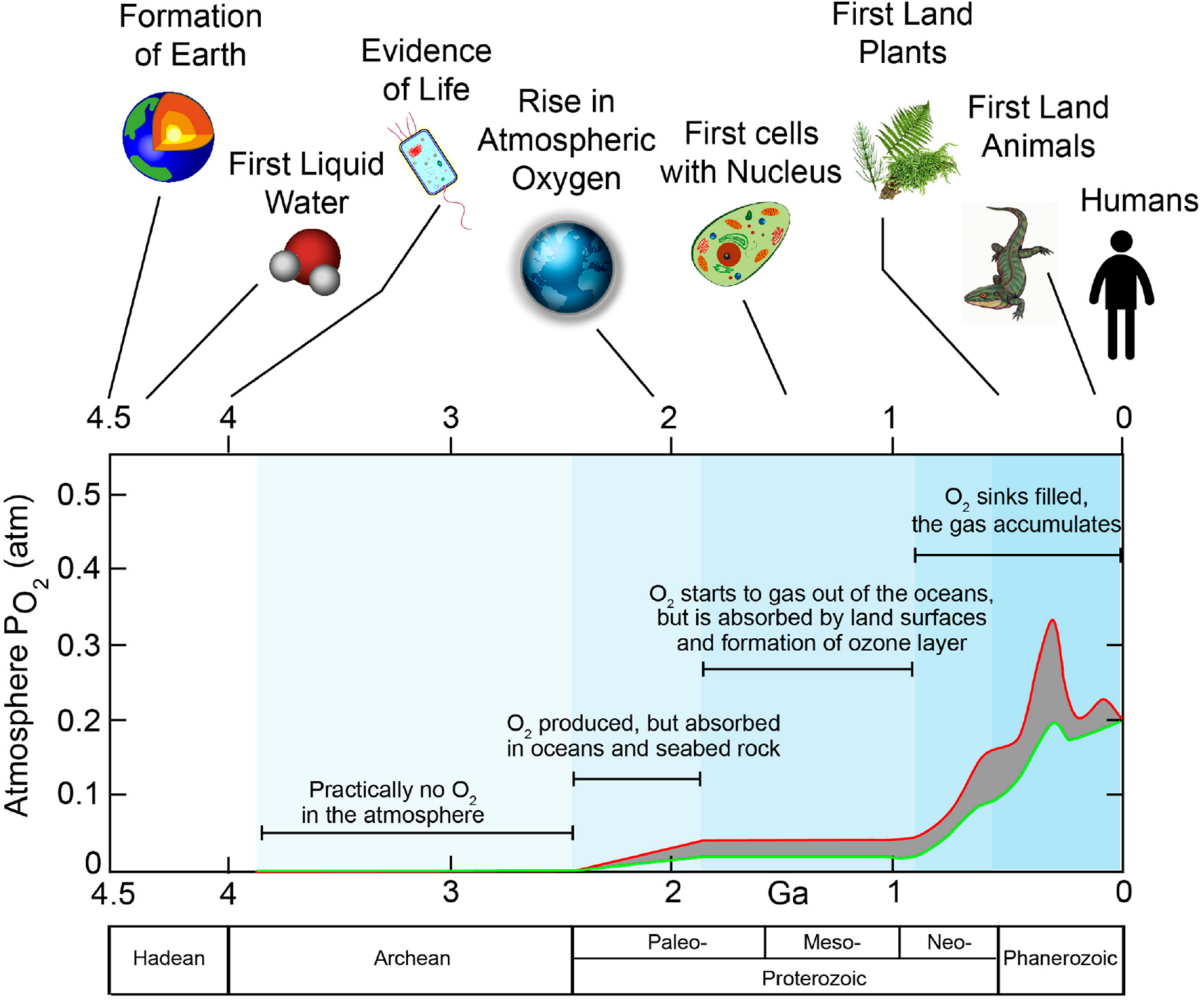
Oxygen build-up in the Earth’s atmosphere on a time scale of billions of years (Ga) and some major events in the development of our planet. The red and green curves denote an upper and lower estimate of the oxygen in the atmosphere. Oxygenic photosynthesis started about ≈ 3.5 Ga ago (Planavsky et al. 2014), the release of O_2_ in the atmosphere ≈ 2.4 Ga ago (Bekker et al. 2004). The present level of O_2_ is ≈ 21% (Holland 2006)

A byproduct of biological water splitting is dioxygen (O_2_) (Fig. 1). Its accumulation over the last billion years has led to the formation of the oxygen-rich atmosphere of our planet (Fischer et al. 2016; Hamilton et al. 2016). It has also caused build-up of the ozone layer in the stratosphere that provides an efficient shield against high-energy UV radiation from the sun, thus protecting and sustaining life on earth. The increase in the concentration of O_2_ in the atmosphere started about two and a half billion years ago (Bekker et al. 2004) and led to the ‘great oxygenation event’ (GOE), a catastrophe for most early life forms on earth which had no defense mechanism to cope with the toxic oxygen, leading to mass extinction. On the other hand, the presence of O_2_ in the atmosphere fostered the development of higher, aerobic life forms, i.e., organisms that evolved to use oxygen for their metabolism. Thus, without the earlier invention of oxygenic photosynthesis, the development of complex life forms with cellular respiration—from simple vertebrates to human beings—would not have taken place. It is, therefore, not only of scientific but also of cultural value for mankind to understand how water oxidation and oxygen release functions in photosynthesis.

We benefit from photosynthesis in many ways. It is our only source of food and supplies us with much of our energy in the form of fossilized photosynthetic material—oil, coal and natural gas. Furthermore, it provides us with valuable natural materials like wood, paper, cotton, peat and biomass in general. Plants also contain many important bioactive materials that are used as drugs, in cosmetics, as natural dyes etc.

A basic understanding of the way how Nature stores energy and, in particular, uses sunlight to split water, could open future pathways to harvest and store the sun’s abundant energy, satisfying the energy demands of society in a sustainable fashion i.e., by an “artificial photosynthesis” (acatech et al. 2018; Chabi et al. 2017; Collings and Critchley 2005; Cox and Lubitz 2013; Cox et al. 2015; Faunce et al. 2013; Gratzel 2005; McKone et al. 2016; Messinger et al. 2014; Nocera 2012, 2017; Wydrzynski and Hillier 2012). In the short review below the current knowledge of the principles of photosynthetic water oxidation in photosystem II of oxygenic organisms are described with special emphasis on the recent X-ray crystallographic data and the results obtained on the electronic structure of the water oxidizing cluster and the water binding and processing derived from advanced EPR techniques in combination with quantum chemical calculations. For a more extensive and detailed description of photosynthetic water oxidation in PS II the reader is referred to some recent reviews (Junge 2019; Pantazis 2018; Shen 2015; Vinyard and Brudvig 2017; Yano and Yachandra 2014).

## The structure of photosystem II and the oxygen-evolving complex

In oxygenic photosynthesis, sunlight is collected by the light-harvesting complexes (LHCs), specialized chlorophyll (and carotenoid) containing proteins, which show a large variation depending on the type of organism (Croce and van Amerongen 2014). The light energy is funneled to a reaction center (RC) where charge separation across the photosynthetic membrane takes place. Two RCs—photosystem I and II (PS I, PS II)—work in tandem to create the redox potential necessary to drive the coupled reactions (Blankenship 2014). This process provides electrons with sufficiently negative potential needed to reduce NADP^+^ to NADPH, which can be considered “bound biological hydrogen”. The electrons for this process originate from water oxidation that takes place in PS II. This process, and subsequent reactions, also generate a proton gradient across the photosynthetic membrane, which is used by the enzyme ATPase to produce ATP from ADP (Junge and Nelson 2015). In a different cell compartment, ATP and NADPH are then used to reduce CO_2_ to carbohydrates in the so-called dark reactions (Calvin–Benson cycle) (Calvin 1962).

Much of our current understanding of the photosynthetic machinery has come from high-resolution crystal structures of its constituents, particular photosystem I and II. The first crystal structure of a bacterial photosynthetic reaction center (BRC) was reported by Deisenhofer, Michel and Huber in the early eighties (Deisenhofer et al. 1985; Michel 1982, 1983)—who subsequently received the Nobel Prize in 1988 for this work. The structure of the simpler BRC provided a model for the functional core of PS I and PS II, in particular the processes of charge separation and progressive radical pair migration. It did not, however, provide any information on the water oxidizing complex (WOC) which is also called the oxygen-evolving complex (OEC), as this component is not found in the BRC that instead uses another electron source.

A crystal structure of a functional oxygen-evolving PS II core complex took almost two decades to be obtained. This was first achieved by the groups of Horst T. Witt and Wolfram Saenger in Berlin who reported the first crystallization of PS II from the cyanobacterium *Synechococcus* (*S.*) *elongatus* (later renamed *Thermosynechococcus* (*T.*) *elongatus*) and obtained a structure with 3.8 Å resolution in 2001 (Zouni et al. 2001). Although this first crystallographic structure provided valuable insight into the arrangement of the protein subunits and the cofactors of PS II it did not allow the positions of the atoms in the OEC, a tetranuclear manganese cluster, to be determined. This is because of radiation damage induced by the intense X-ray beam used for X-ray diffraction (XRD) data collection in modern synchrotrons. X-ray absorption spectroscopy (XAS) experiments with varying beam intensity by Yano and coworkers have shown photoreduction of the oxidized Mn ions, disintegration of the complex and blurring of the electron density associated with the cluster (Yano et al. 2005). Before the advent of a PS II crystal structure with sufficient resolution attempts have been made to obtain models of the tetranuclear Mn cluster from spectroscopic experiments, in particular from XAS spectroscopy (Yachandra et al. 1996; Yano et al. 2006). In 2004 the group of James Barber (Imperial College London) presented an improved structure of PS II from *S. elongatus* at 3.5 Å resolution (Ferreira et al. 2004). Based on their refined data, including anomalous diffraction to identify the Ca, and also on previous EXAFS data (Robblee et al. 2001) the authors proposed a heterometallic cubane-type structure of the OEC, which contained a Mn_3_Ca unit along with a more distant dangler Mn. Such a “dangler model” had been proposed earlier by David Britt’s group (UC Davis) based on constraints from EPR and ENDOR experiments and the distances obtained from EXAFS (Peloquin and Britt 2001; Peloquin et al. 2000). Finally, in 2011 the groups of Jian-Ren Shen (Okayama, Japan) and Nobuo Kamija (Osaka, Japan), who had reported a low resolution structure of PS II in 2003 (Kamiya and Shen 2003) published a significantly improved structure from *Thermosynechococcus* (*T.*) *vulcanus* at 1.9 Å resolution, in which the atomic arrangement of the ions in the OEC was finally resolved (see below) (Umena et al. 2011). Subsequent theoretical studies showed that the actual oxidation state of the OEC in this structure was a mixture of overreduced states, not present in the catalytic cycle (Galstyan et al. 2012; Luber et al. 2011). The Shen group also succeeded in obtaining a structure with reduced radiation damage using femtosecond (fs) pulsed X-ray crystallography with a free electron laser (XFEL) (Suga et al. 2015). More recently, time resolved XFEL measurements (serial femtosecond crystallography—SFX) near ambient temperature has emerged as an important tool for the investigation of the various states of the catalytic cycle of the OEC (see below) (Kern et al. 2012, 2013, 2014, 2018; Kupitz et al. 2014; Suga et al. 2015, 2017).

**Fig. 2:**
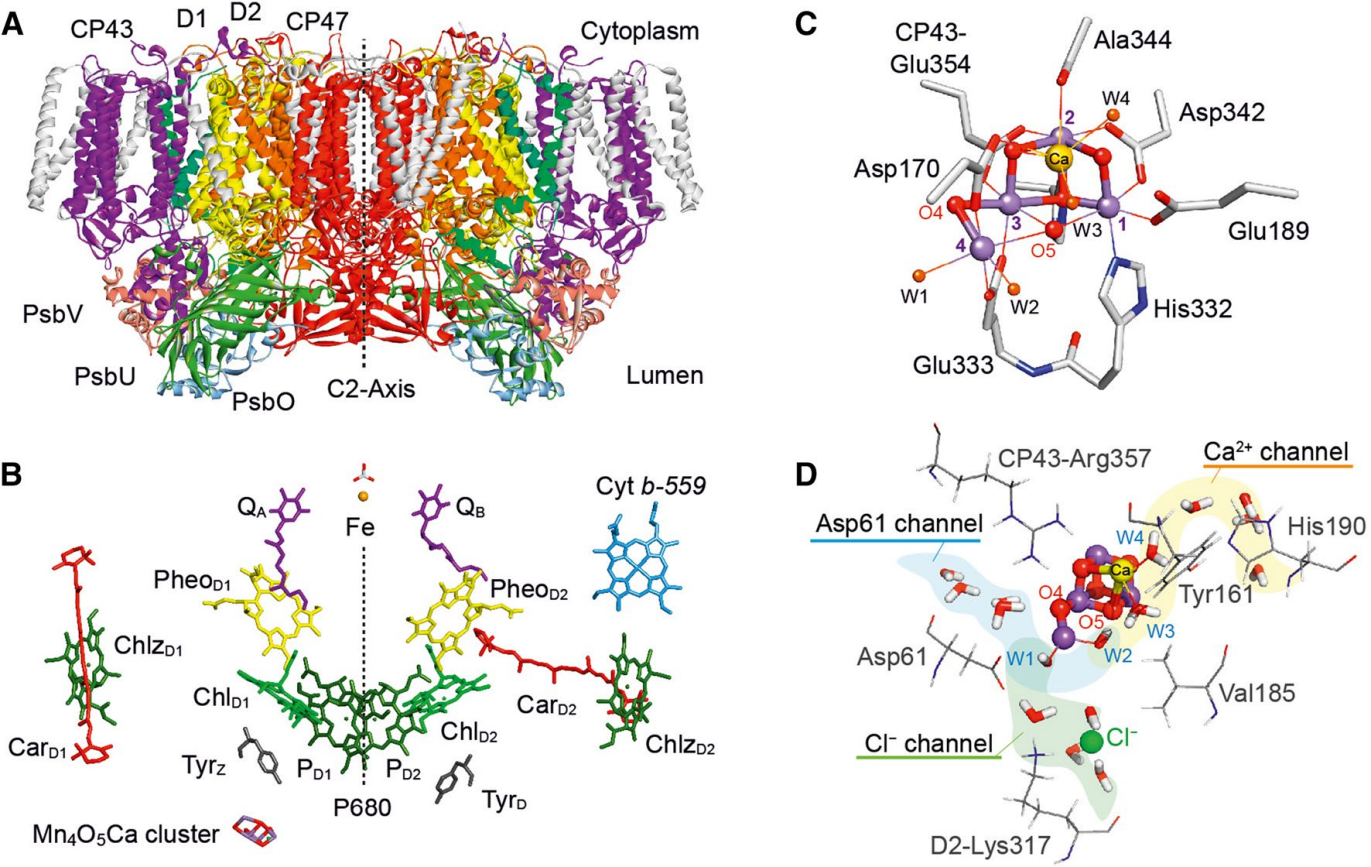
X-ray crystallographic structure of photosystem II (PS II) from *T. vulcanus*. **A** View of the dimeric protein (molecular weight of the dimer ≈ 700 kDa); the two monomers are related to each other by a C2 axis. The most important subunits are indicated, CP43, CP47, in which the core antenna pigments are located; D1 and D2, which bind all pigments of the reaction center and all cofactors of the electron transport chain, and the small subunits PsbO, PsbU and PsbV, which stabilize the water splitting unit. The D1 protein (yellow) holds the active branch cofactors and the Mn cluster, the D2 protein (orange) the cofactors of the second pigment branch. **B** Pigment arrangement in one PS II monomer; the two branches are related by a pseudo-C2 axis (dotted line). Shown are the primary donor P680 (four chlorophylls: P_D1_, P_D2_, Chl_D1_, Chl_D2_), the two pheophytins (Pheo_D1_, Pheo_D2_), the two plastoquinones (*Q*_A_, *Q*_B_), and the non-heme iron Fe. Additional chlorophylls (Chlz) and carotenoids (Car), as well as two redox-active tyrosines (Tyr_Z_, Tyr_D_) are also indicated. Next to the active branch (D1) and close to Tyr_Z_/P680, the Mn_4_O_5_Ca cluster is located. **C** Structure of the water oxidizing Mn_4_O_5_Ca cluster in PS II with four Mn ions (Mn1 to Mn4, purple) and one Ca (yellow), bridged by oxygen ligands (red). Three Mn ions (1 to 3) and the Ca form a distorted cube bridged by oxygen ligands, the fourth Mn (Mn4) is dangling. Mn4 and the Ca carry two water molecules each (W1 to W4, orange). The coordination of the metal ions by amino acid ligands is also shown; for further details see (Suga et al. 2015; Umena et al. 2011). **D** Water channels leading to the OEC: Three channels have been localized. Note that also the essential chloride ion is shown in this picture (Suga et al. 2015; Umena et al. 2011). A second Cl^−^ is found farther away from the OEC. It has been pointed out that the channels could be multi-functional

In Fig. 2 the dimeric PS II protein is shown (Suga et al. 2015), located in the thylakoid membrane. Each monomer is made up of 20 protein subunits (in cyanobacteria). A few important ones are indicated in Fig. 2A. In Fig. 2B the cofactor arrangement in the D1/D2 subunits is shown—comprising chlorophylls (P_D1_, P_D2_, Chl_D1_, Chl_D2_, Chlz_D1_, Chlz_D2_), carotenoids (Car_D1_, Car_D2_), pheophytins (Pheo_D1_, Pheo_D2_), plastoquinones (Q_A_, Q_B_), non-heme iron (Fe) and also the WOC or OEC (Fig. 2C). This cubane-like cluster is bound in the PS II protein and comprises four Mn ions and one Ca ion linked by five oxygen bridges (six in the last stage of the catalytic cycle). Remarkably, it also binds four water molecules, two at the Ca and two at the dangling Mn4. The cluster is connected to the protein surface via specific channels for the efficient uptake of water, and the release of protons and molecular oxygen (Bondar and Dau 2012; Gabdulkhakov et al. 2009; Ho and Styring 2008; Linke and Ho 2014; Murray and Barber 2007; Retegan and Pantazis 2017; Saito et al. 2012; Umena et al. 2011; Vassiliev et al. 2012). For each PS II monomer about 1300 water molecules have been detected in the crystal structure (Umena et al. 2011); they are mostly found at the outside membrane region but quite a few are located inside of the PS II protein. The water access channels to the Mn cluster are shown together with a few key amino acid ligands in Fig. 2D. The first channel is via the Asp 61 (shaded blue) the second via the Ca (shaded yellow) and a possible third one via the Cl^−^ binding site (green). In all channels several water molecules have been found in the crystal structure (Ferreira et al. 2004; Gabdulkhakov et al. 2009; Ho and Styring 2008; Murray and Barber 2007; Saito et al. 2012; Umena et al. 2011; Vassiliev et al. 2012). Cl^−^ depletion inhibits water oxidation; exchange of Cl^−^ with Br^−^ or I^−^ slows the water oxidation kinetics (Damoder et al. 1986; Ono et al. 1987). The effect of these halogen anions is probably related to charge effects in the protein and proton egress.

## Basic function of photosystem II: kinetics and energetics of water oxidation

Light excitation of the reaction center of PS II, a multi-pigment assembly of four chlorophyll-*a* and two pheophytin-*a* molecules, leads to primary charge separation on a picosecond timescale. This is a single electron transfer process resulting in a charge-separated radical pair (RP) state comprising the radical cation P680^·+^ and the radical anion Pheo^·−^ (Cardona et al. 2012). During primary charge separation, the positive charge is initially localized on the accessory chlorophyll Chl_D1_ and it is stabilized by subsequent transfer on P_D1_ (see Fig. 2A) (Groot et al. 2005; Holzwarth et al. 2006; Kammel et al. 2003). The primary acceptor Pheo is the pheophytin pigment bound to the D1 protein (Holzwarth et al. 2006). The oxidation potential of P680^·+^ is estimated to + 1.2 to + 1.3 V, the highest known in biology (Rappaport et al. 2002). The primary RP P680^·+^Pheo^·−^ is stabilized by subsequent electron/hole transfer steps. This is important as the radical cation can, in principle, oxidize neighboring chlorophylls, and the protein itself. To suppress both side and back reactions, nature has placed secondary donor/acceptors in close proximity to the primary RP. On the donor side of the protein (lumen), it is the redox-active tyrosine residue D1-Tyr161 (known as Tyr_Z_ or *Y*_z_) next to P680^·+^ that is able to quickly donate an electron to this species on a nanosecond (20–250 ns) time scale, thereby reducing the radical cation to its initial state P680. The tyrosine radical cation *Y*_z_^·+^ is stabilized by losing a proton to the neighboring D1-His190 in a reversible fashion, forming a neutral tyrosine radical (*Y*_z_^·^) (Berthomieu et al. 1998; Chu et al. 1995; Saito et al. 2011). *Y*_z_^·^ in turn oxidizes the adjacent Mn cluster in 40–1600 µs dependent on the *S*_*i*_ transition (Babcock et al. 1976; Brettel et al. 1984; Dekker et al. 1984a; Haumann et al. 2005a; Karge et al. 1997; Klauss et al. 2012a; Noguchi et al. 2012; Rappaport et al. 1994; Razeghifard and Pace 1997). On the acceptor side of the protein (cytoplasm), the acceptor Pheo^·−^ passes its electron to the bound plastoquinone molecules (to *Q*_A_ and subsequently to *Q*_B_). Reducing equivalents derived from this process are temporarily stored as reduced plastoquinol (*Q*_B_H_2_), a mobile electron carrier, generated after two light absorption/charge separation and protonation events.

Other light-induced processes that occur in PS II are equally dangerous, for which nature has not found a perfect solution to circumvent them. This is the creation of triplet states, e.g., of the chlorophylls in the core antenna via intersystem crossing or in the RC via (triplet) radical pair recombination. While carotenoid molecules are present in PS II to suppress (quench) triplet chlorophyll formation, these states can still react with triplet oxygen (^3^O_2_) formed in the water oxidation process yielding singlet oxygen (^1^O_2_) (Macpherson et al. 1993). This very aggressive species can then react with the pigments and/or the protein, resulting in damage of the D1 protein subunit of the PSII supercomplex. The production of such reactive oxygen species (ROS) (Vass 2012) limits the lifetime of PS II to only 30 min under normal light conditions. Owing to its remarkable efficiency (the turnover time of the OEC is ≈ 2 ms, that of the whole PS II ≈ 10 ms)(Vinyard and Brudvig 2017), it performs more than 10^5^ reaction cycles before it must be replaced. Fortunately, all organisms performing oxygenic photosynthesis have developed an efficient repair mechanism for the PS II supercomplex by the discrete replacement of the D1 protein (Nixon et al. 2010).

PS II thus acts as a water:plastoquinone oxidoreductase (Wydrzynski and Satoh 2005). The products are molecular (triplet) oxygen (^3^O_2_), protons and reduced plastohydroquinone. The light-induced process in PS II is very efficient, with a quantum yield of over 90% and an energy efficiency of about 20%. This value is, however, strongly attenuated when all the subsequent processes are considered, such that the total biomass generated often contains only less than 1% of the original light energy input (Blankenship et al. 2011; Michel 2008).

**Fig. 3:**
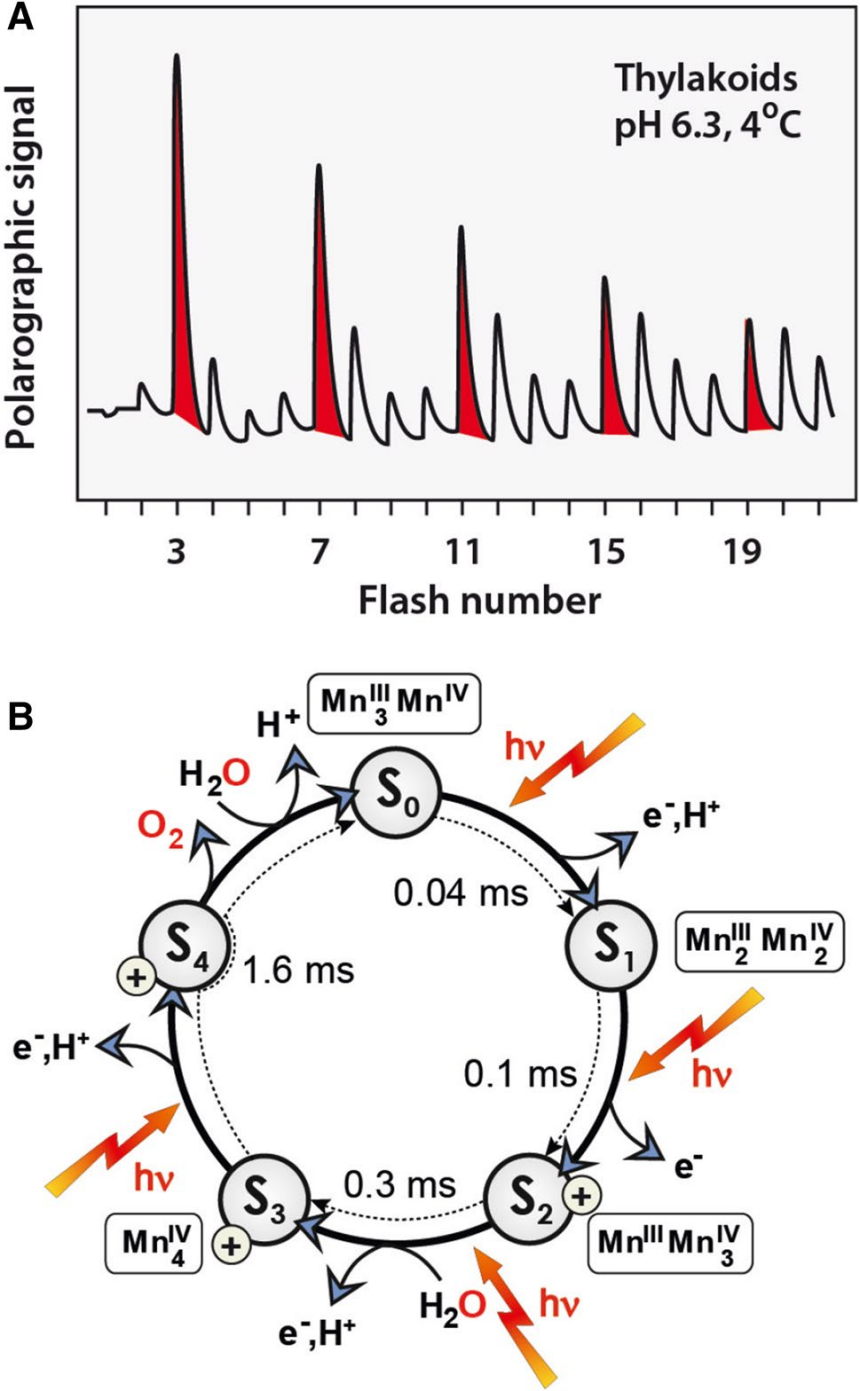
**A** Release pattern of molecular oxygen (O_2_) measured polarographically following successive light flashes of spinach thylakoids at 4 °C (Messinger and Renger 2008). Note that O_2_ release follows a 4-flash pattern (the starting dark stable state is *S*_1_). The original experiment was performed by Pierre Joliot as early as 1969 (Joliot et al. 1969). **B** Water oxidation cycle (Kok cycle) (Kok et al. 1970) detailing the five basic *S* states (*S*_0_ to *S*_4_), the light-induced 1e^−^ oxidation steps and the proton release pattern (Dau and Haumann 2008), the uptake of the two substrate waters (Hillier and Wydrzynski 2008) and the Mn oxidation states (Krewald et al. 2015) (vide infra). The reaction times for the single electron oxidation steps are also indicated (Klauss et al. 2012a). Note that here the “*S*” stands for “state” and not for the electron spin quantum number *S*

It was discovered by Pierre Joliot almost half a century ago that PS II releases oxygen after four consecutive light flashes (Joliot et al. 1969) (Fig. 3A). This shows that four light-induced trans-membrane charge separation events are necessary before one O_2_ molecule is formed and released. This result is in agreement with the fact that the oxidation of two water molecules to produce a single O_2_ molecule is a four-electron process,

1 $${\text{ 2H}}_{ 2} {\text{O }} \to {\text{ O}}_{ 2} + {\text{ 4H}}^{ + } + {\text{ 4e}}^{ - } ,$$

whereas the charge separation across the membrane is a one-electron process. The oxidizing equivalents for the water oxidation reaction are stored transiently by the tetranuclear manganese cluster, an insight that came from Kok et al. (1970). His so-called Kok cycle (S-state cycle) of water oxidation, shown in Fig. 3B, comprises five distinct states, *S*_0_ to *S*_4_, which differ by the number of oxidizing equivalents transiently stored in the cofactor, as indicated by their subscript. The cycle is driven by four light absorption events (hν), by which electrons are withdrawn from the metal ions and protons are released to avoid the accumulation of positive charges and thus prevent a “coulombic explosion” of the cluster. O_2_ is released in a concerted reaction in the last reaction step with an equilibrium constant *k* > 1.0 × 10^7^ (Nilsson et al. 2016). Kinetic measurements showed that the reaction times lie in the micro- to millisecond range (40 µs to 1.6 ms) (Klauss et al. 2012a), see Fig. 3B; the complete cycle turns over in about 2 ms. The kinetics of the proton release, which follow a 1:0:1:2 pattern (or 1:0:1:1:1) have also been determined, see (Dau and Haumann 2007; Klauss et al. 2012a, 2015; Schlodder and Witt 1999; Suzuki et al. 2009). The (alternating) electron and proton release led to an extended catalytic cycle for the water oxidation reaction in PS II with nine states that differ in their net electron and proton count (Klauss et al. 2012a, 2015; Perez-Navarro et al. 2016).

In this sequence, Y_Z_ promotes both electron and proton transfer in the catalytic cycle displaying a dual function (Bovi et al. 2013; Klauss et al. 2012b; Perez-Navarro et al. 2016). Between *S*_*i*_ and *S*_*i*+1_ states (for *i* = 0–3) the short-lived S_i_Y_Z_˙ intermediates exist. Studying these intermediates is critical for understanding the role of *Y*_Z_˙ in water oxidation and especially in proton removal from the Mn_4_O_x_Ca cluster (Boussac et al. 2008; Havelius et al. 2010; Nugent et al. 2002; Peloquin et al. 1998; Petrouleas et al. 2005; Retegan et al. 2014; Styring et al. 2012).

**Fig. 4:**
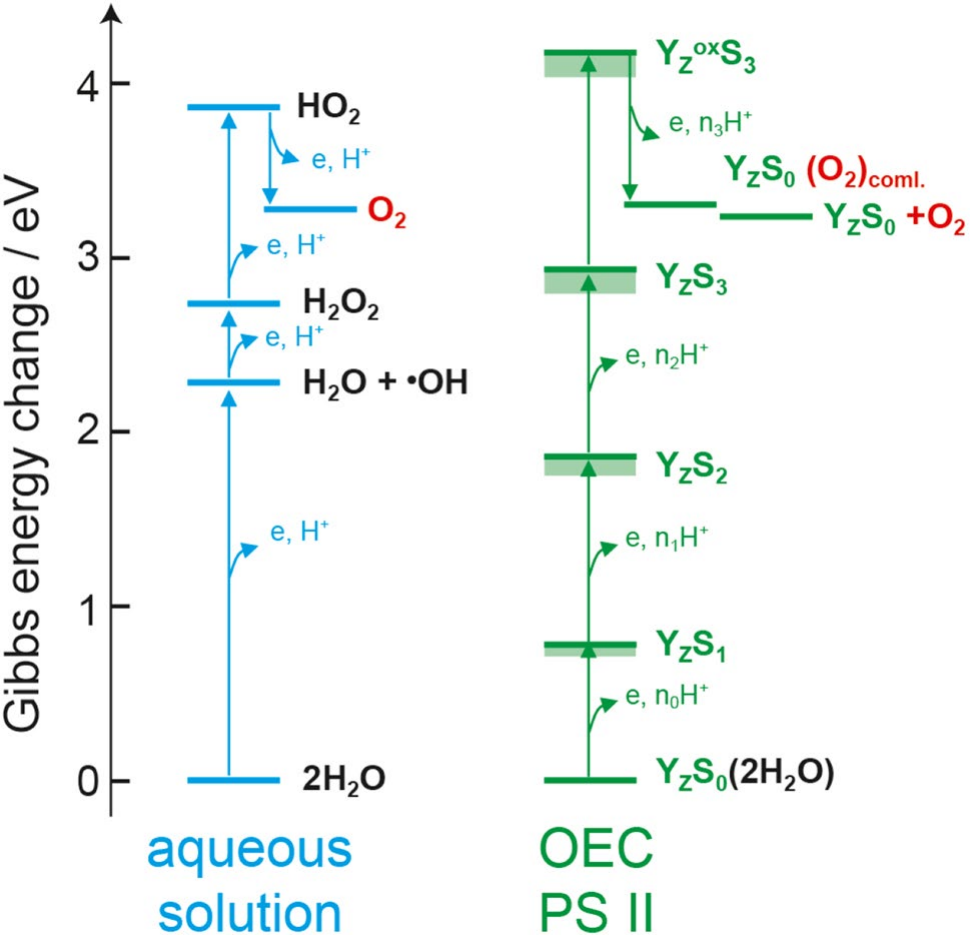
Gibbs energy (in eV) required to oxidize water stepwise in aqueous solution (left) and in the OEC of PS II (right). In particular the removal of the first electron from water (left) requires much energy (> 2 eV) that cannot be provided in a biological system. Thus, in the OEC, water is not oxidized by subsequent single electron removal from substrate water. Instead, it is the Mn cluster that is oxidized by four successive oxidation events; the two attached substrate water molecules release the protons (for charge neutrality), and O_2_ is released only in the last step after O–O bond formation in a concerted reaction. Thereby, high-energy steps are avoided and the redox process is leveled. Figure adapted from (Messinger and Renger 2008)

There are three interlinked reasons why the Mn_4_O_x_Ca cluster functions in this way: (i) The energetic cost is significantly lowered by first storing oxidizing equivalents and then performing the four-electron chemistry; (ii) this sequence avoids the formation of reactive oxygen species (ROS) as intermediates resulting from partial oxidation of substrate water, which are highly reactive and can destroy the PS II protein complex; and (iii) proton release during charge accumulation leads to charge neutrality so that about the same oxidizing potential (oxidant P680^·+^) can be used for all Mn oxidation events. This is illustrated by the scheme in Fig. 4 (Messinger and Renger 2008). Here the Mn ions (and/or their ligands) are oxidized *four* times by the neighboring tyrosine Y_Z_^·^ triggered by *four* charge separation events in PS II before the cluster removes *four* electrons from two bound water molecules in a concerted reaction leading to O_2_ release and regeneration of the original starting state of the Mn_4_O_x_Ca cluster. The Mn cluster thus acts as an interface and storage device between the very fast light reaction (ps time scale) and the slow catalytic reaction (ms time scale) of the 4-electron water oxidation chemistry, bridging a kinetic gap of nine orders of magnitude.

## The electronic structure of the Mn_4_O_x_Ca cluster

A detailed understanding of the catalytic process of water oxidation in PS II requires knowledge of the electronic structure, i.e., the distribution of the electrons in the cluster, in all consecutive reaction steps. The oxidation and spin states of the Mn ions, representing the total number and configuration of electrons in the Mn valence orbitals, give a basic description thereof. These together with the magnetic interactions between the spin-bearing Mn ions, depending to a large part on the metal ligands, provide a comprehensive picture of the respective electronic state, which governs the chemical and catalytic properties of each S state. Thus, the spin states provide information about how the structure of the cofactor evolves during the *S*-state cycle, for a recent review see (Krewald et al. 2016).

An experimental method to investigate the electronic structure of transition metal complexes is electron paramagnetic resonance (EPR) spectroscopy (Goldfarb and Stoll 2018; Schweiger and Jeschke 2001; Weil and Bolton 2007). It exploits a fundamental property of matter, which is that unpaired electrons have an intrinsic angular momentum (spin), which can be excited by microwave radiation in a magnetic field. The unpaired electron spin also interacts with other electron and nuclear spins as well as with local electric field gradients, making it a sensitive reporter of its chemical environment. It is thus analogous to other magnetic spectroscopies such as nuclear magnetic resonance (NMR). Since the Mn ions are open-shell species, i.e., exhibit orbitals with single electron occupancy, whereas most of the electrons of the protein and solvent surrounding are paired, the EPR signals of the Mn_4_O_x_Ca cluster can be detected selectively. It has also been shown that *all* S states in the Kok cycle can be trapped (except for the elusive *S*_4_ state) and that *all* exhibit paramagnetism (Haddy 2007).

**Fig. 5:**
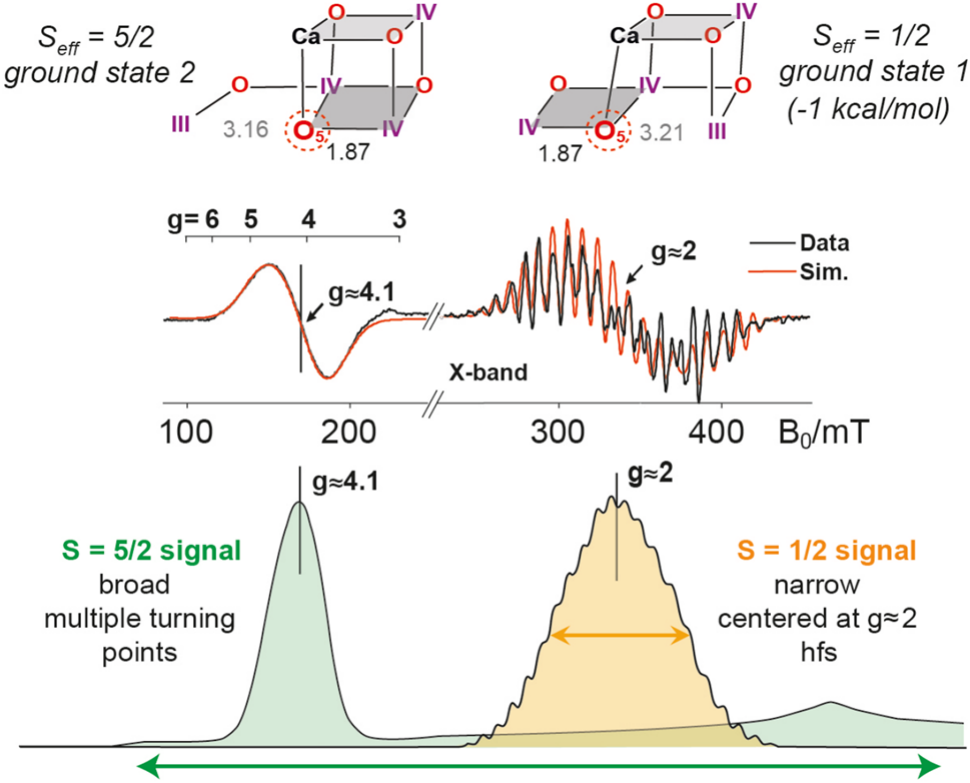
Top: DFT-optimized structures of the Mn_4_O_5_Ca cluster in the S_2_ state (distances given in Ångström). Note that the “closed cubane” (left) and “open cubane” (right) structures have almost the same energy but different positions of the Mn^III^ ion (Mn4 vs. Mn1, respectively) and different total (effective) spin ground states. This explains the two EPR signals observed for this state (shown below as first derivative X-band EPR spectra), with *g* = 4.1 (*S*_eff_ = 5/2; no hyperfine structure) and *g* = 2 (*S*_eff_ = ½; multiline signal). The bottom trace shows simulations of the two EPR absorption signals. Figure modified from (Krewald et al. 2016; Pantazis et al. 2012)

The first EPR signal observed by Charles Dismukes in 1981 (Dismukes and Siderer 1981) came from the trapped *S*_2_ state. Centered at *g* ≈ 2 it included over 20 resolved lines and was thus called “multiline signal” (see Fig. 5). The structure is due to the coupling of the electron spin with the four ^55^Mn nuclei (*I* = 5/2) via the electron nuclear hyperfine interaction. The EPR signal represents an effective low-spin ground state of *S*_eff_ = ½ (Bencini and Gatteschi 1990). The spin state depends on the oxidation state of the Mn ions, their geometry and in particular on the (bridging) ligands which connect the metal ions. These mediate antiferromagnetic or ferromagnetic exchange interactions between the Mn ions leading to either a low-spin state, minimizing the number of unpaired electrons, or a high-spin state, maximizing the number of unpaired electrons. The studies of the groups of Ono and Kusunoki on oriented PS II membranes and theoretical investigations (Hasegawa et al. 1998, 1999) and subsequent advanced EPR studies from the Britt (UC Davis) (Britt et al. 2000, 2004; Peloquin et al. 2000) and Lubitz (MPI Mülheim) laboratories (Cox et al. 2011; Kulik et al. 2005a, b, 2007; Lohmiller et al. 2012, 2014; Su et al. 2011) further constrained all four ^55^Mn hyperfine tensors in the *S*_2_ state and allowed the spin coupling in the tetranuclear manganese cluster to be interrogated. These data together with results collected on the *S*_0_ state, which also resolves a multiline signal (Ahrling et al. 1997; Kulik et al. 2005b, 2007; Lohmiller et al. 2017; Messinger et al. 1997), and density functional theory calculations allowed the local oxidation states and set of coupling pathways to be determined (Ames et al. 2011; Krewald et al. 2013, 2015, 2016; Pantazis et al. 2012, 2009). The oxidation state assignment for the S_2_ state, which comes from this analysis, is shown in Fig. 5 (top), i.e., Mn_4_(III, IV, IV, IV). According to the low oxidation scheme *S*_0_ includes a Mn^II^ (Kolling et al. 2012; Pace et al. 1991; Terrett et al. 2016), but this was excluded by the ^55^Mn ENDOR experiments and analysis of the S_0_ state (Kulik et al. 2005b, 2007; Lohmiller et al. 2017). The reader interested in details on how the electronic configuration of a polynuclear Mn cluster, especially the Mn_4_CaO_5_, can be probed by EPR spectroscopy and double resonance techniques in relation with calculations of magnetic properties by quantum mechanical methods is referred to the following review (Lohmiller et al. 2013). Additional support for the high oxidation state scheme has come from the detection of ^14^N (*I* = 1), ^2^H (*I* = 1) and ^13^C (*I* = ½) hyperfine couplings of the *S*_2_ state using ESEEM and ENDOR techniques performed by the group of R. D. Britt (Britt et al. 1989; Marchiori et al. 2018; Perez-Navarro et al. 2013; Oyala et al. 2015; Stich et al. 2011; Stull et al. 2010); see also (Lohmiller et al. 2017; Perez-Navarro et al. 2013). X-ray spectroscopies also agree with this assignment (Chatterjee et al. 2016; Zaharieva et al. 2016a, b). These studies rule out the alternative lower oxidation state models for the manganese cluster (Kolling et al. 2012; Pace et al. 1991) that are, however, still discussed in the literature (Chen et al. 2018; Terrett et al. 2016).

In the S_2_ state, in addition to the low-spin *S*_eff_ = ½ form showing the characteristic multiline signal around *g* ≈ 2, the cluster can also be found in a high-spin *S*_eff_ = 5/2 state under certain conditions. This is evident from an EPR signal around *g* = 4.1 (see Fig. 5), which has been observed earlier by several research groups (Boussac et al. 1996; Casey and Sauer 1984; Haddy et al. 2004; Zimmermann and Rutherford 1984). Pantazis et al. could show computationally that the two electronic structures are a direct consequence of two different spatial conformations of the manganese cluster, namely a “closed cubane” (*S*_eff_ = 5/2) and an “open cubane” (*S*_eff_ = ½) form, which have almost the same energy (Bovi et al. 2013; Isobe et al. 2012; Pantazis et al. 2012). In these two structures one of the oxygen bridges, O5, is occupying different positions. In turn this also led to a change of the Mn^III^ position (Jahn–Teller ion) and thus of the open coordination site of the cluster resulting in a change of the precise electronic properties of the cofactor. EXAFS data also support this model (Chatterjee et al. 2019). This shows that the effective spin *S*_eff_ is a crucial parameter for describing a particular state of the cluster and assigning it a spatial structure. It is postulated that O5 has not a fixed position but toggles between two structures in the dynamic *S*_2_ state, which is important for the water binding and the catalytic mechanism (see below).

Modifications of the Mn_4_O_x_Ca cluster, such as exchange of Ca^2+^ with Sr^2+^ (Boussac and Rutherford 1988; Boussac et al. 2015; Chu et al. 2000; Cox et al. 2011; Koua et al. 2013; Yachandra and Yano 2011), its complete removal (Boussac et al. 1989; Lohmiller et al. 2012; Styring et al. 2003; Vrettos et al. 2001b) and the binding of small molecules (Beck et al. 1986; Perez-Navarro et al. 2013; Oyala et al. 2015; Oyala et al. 2014; Retegan and Pantazis 2016; Su et al. 2011), provided further insight into the conformation and number of ligands of individual Mn ions (coordination geometry) and substrate access in the *S*_2_ state. Upon Ca depletion water oxidation functionality is lost, but the *S*_2_ state (*S*_2_′) is still formed (Boussac et al. 1989, 1990). Interestingly, the EPR and ^55^Mn ENDOR data of S_2_′ show that the calcium has no substantial effect on the magnetic parameters of the S_2_ state; this ion is thus not crucial for maintaining the electronic structure of the tetranuclear Mn cluster (Lohmiller et al. 2012). Instead, it might serve as stage for the delivery of water molecules to the reaction site (Nakamura et al. 2016; Service et al. 2014; Ugur et al. 2016), affect the function of *Y*_Z_, (Miqyass et al. 2008; Retegan et al. 2014) and introduce some structural flexibility allowing the cofactor to toggle between the different motifs of the open and closed cubane structures (see Fig. 5). The interactions of small molecules which mimic the substrate water (methanol, ammonia) also support this potential role for Ca^2+^. These molecules associate with the Ca^2+^ and Asp61 water channels that lead to the Mn_4_O_x_Ca cofactor (Marchiori et al. 2018; Navarro et al. 2013; Oyala et al. 2014; Retegan and Pantazis 2016; Schraut and Kaupp 2014) (see Fig. 2D). In addition, both of these molecules reduce turnover efficiency; these results implicate that at least one (or possibly both) of these channels are involved in substrate delivery.

**Fig. 6:**
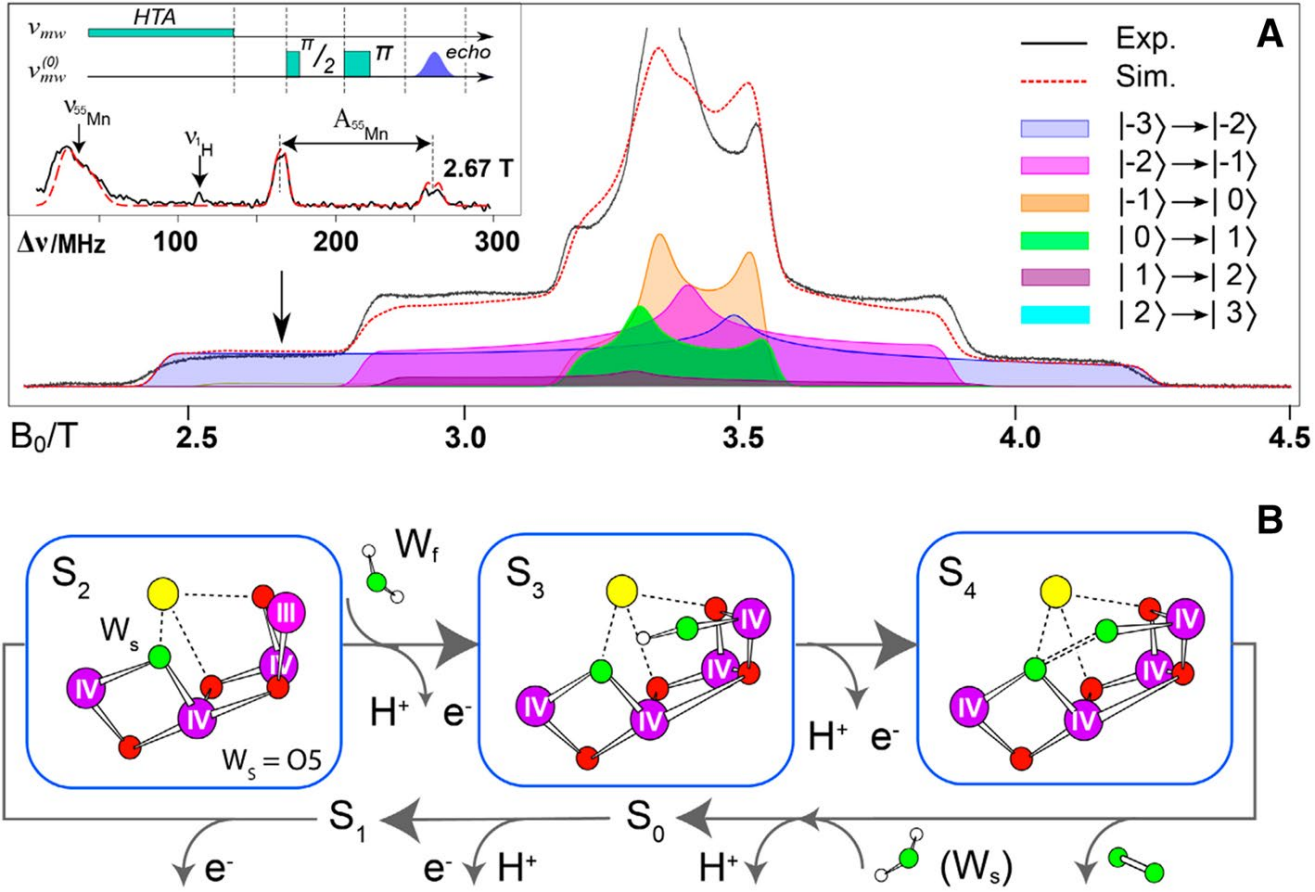
**A** W-band EPR spectrum of the OEC in the *S*_3_ state in PS II of *T. elongatus* (*S*_3_–*S*_1_ light–dark difference spectrum) characteristic of an *S*_eff_ = 3 ground state. The red-dashed line shows a simulation with *g* and fine structure (zero field splitting) parameters D and E/D; the shaded curves show the contributions of the allowed EPR transitions to the simulated powder spectrum, the color code is indicated. As insert a ^55^Mn EDNMR spectrum is shown obtained at the EPR position indicated by the arrow. The used EDNMR pulse sequence is given above. Figure modified from (Cox et al. 2014). **B** Structure of the manganese cluster proceeding through the *S*_2_ → S_3_ → *S*_4_ states including water *W*_f_ binding, proton release and O_2_ formation as suggested by magnetic spectroscopy

The EPR measurements have been extended to the S_3_ state. These results are of particular importance since this is the last metastable state prior to O–O bond formation and O_2_ release. The EPR signal of the S_3_ state has first been reported to originate from a ground state with an integer spin of *S*_eff_ = 3 by the groups of Petrouleas (Sanakis et al. 2008) and Boussac (Boussac et al. 2009). Recent pulse high field (W-band) EPR experiments by Cox et al. (see Fig. 6A) corroborated this assignment and directly proved that the effective spin state is indeed *S*_eff_ = 3 by measuring the Rabi oscillations via a spin nutation experiment (Cox et al. 2014). In addition ^55^Mn ELDOR-detected-NMR (EDNMR) experiments at W-band could successfully be performed showing that in the S_3_ state all Mn ions are in the Mn^IV^ state Mn_4_ (IV,IV,IV,IV) and octahedrally coordinated. Thus, no ligand oxidation takes place as previously proposed (Kawashima et al. 2018; Messinger et al. 2001). The results also show that a sixth ligand is binding to the open coordination site of the Mn^III^ present in the S_2_ state when it is oxidized. This ligand is most probably the second substrate water molecule which also loses a proton in the *S*_2_ → *S*_3_ transition. Data from XAS, XES and FTIR further support these finding (Noguchi 2008; Sakamoto et al. 2017; Zaharieva et al. 2016a, b). Mn oxidation in the S_2_ to S_3_ transition has been proposed by Dekker (Dekker et al. 1984a, b) and Ono (Ono et al. 1992) and substantiated by Dau and coworkers (Haumann et al. 2005a, b). It has been included in mechanistic models (Pecoraro et al. 1998; Vrettos et al. 2001a) and has also theoretically been supported by Siegbahn (Siegbahn 2009). Very recently, SFX data have shown additional electron density acquired during the *S*_2_ to *S*_3_ transition, consistent with the binding of a light atom (e.g., an oxo or hydroxyl ligand) in the manganese cluster (Kern et al. 2018; Suga et al. 2017).

The magnetic resonance experiments described above together with theoretical calculations allowed a reliable characterization of the *S*_0_, *S*_2_ and *S*_3_ states with respect to oxidation and spin states of individual ions and their spin coupling in the tetranuclear Mn cluster summarized by Krewald et al. (2015, 2016). Together with information about binding of the two substrate water molecules described below spatial models of the S states could be obtained that form the basis for developing a catalytic mechanism for the OEC (Fig. 8).

## Identification of the substrate water molecules in water oxidation

Knowledge of the binding and dynamics of the substrate water molecules is crucial for formulating the reaction mechanism of photosynthetic water oxidation. Candidates for substrate waters, besides the four H_2_O/OH^−^ molecules (Fig. 2C) directly attached to the cluster (Ames et al. 2011; Suga et al. 2015; Umena et al. 2011), are oxygen bridges within the cluster as well as possible water molecules binding at a later point in the catalytic cycle. Apart from X-ray diffraction spectroscopic techniques are used to investigate water binding, in particular EPR (Cox et al. 2013b; Fiege et al. 1996; Kawamori et al. 1989; McConnell et al. 2012; Nagashima and Mino 2013; Rapatskiy et al. 2012) and vibrational (infrared) spectroscopy (Debus 2014, 2015; Kim et al. 2018; Kim and Debus 2017; Nakamura et al. 2016; Noguchi 2008; Sakamoto et al. 2017; Suzuki et al. 2008). These are used in conjunction with time-resolved mass spectrometry (MIMS) which detects the uptake of H_2_^18^O labeled water into the product O_2_; for a comprehensive review on the identification of possible water substrates by MIMS see (Cox and Messinger 2013). MIMS experiments showed that the two substrate water molecules exchange at different rates in all of the S states (Hillier and Wydrzynski 2000, 2004, 2008). The more slowly exchanging water (*W*_s_) has an exchange rate of the order of seconds (Messinger et al. 1995) while the faster exchanging water (*W*_f_) has an exchange rate of milliseconds (Hillier et al. 1998) (see Fig. 3B). The observation of two rates implies that the two substrates bind at two chemically distinct sites. They also demonstrate that the O–O bond is not formed until the (*S*_4_) state is reached. Interestingly, it was recently observed that during the lifetime of the *S*_3_*Y*_Z_˙ state, that lies between *S*_3_ and the elusive S_4_ state while the *Y*_Z_˙ radical exists, the water exchange is slowing down, which may suggest that the two oxygen species are “arrested” in a bonding formation (Nilsson et al. 2014).

**Fig. 7:**
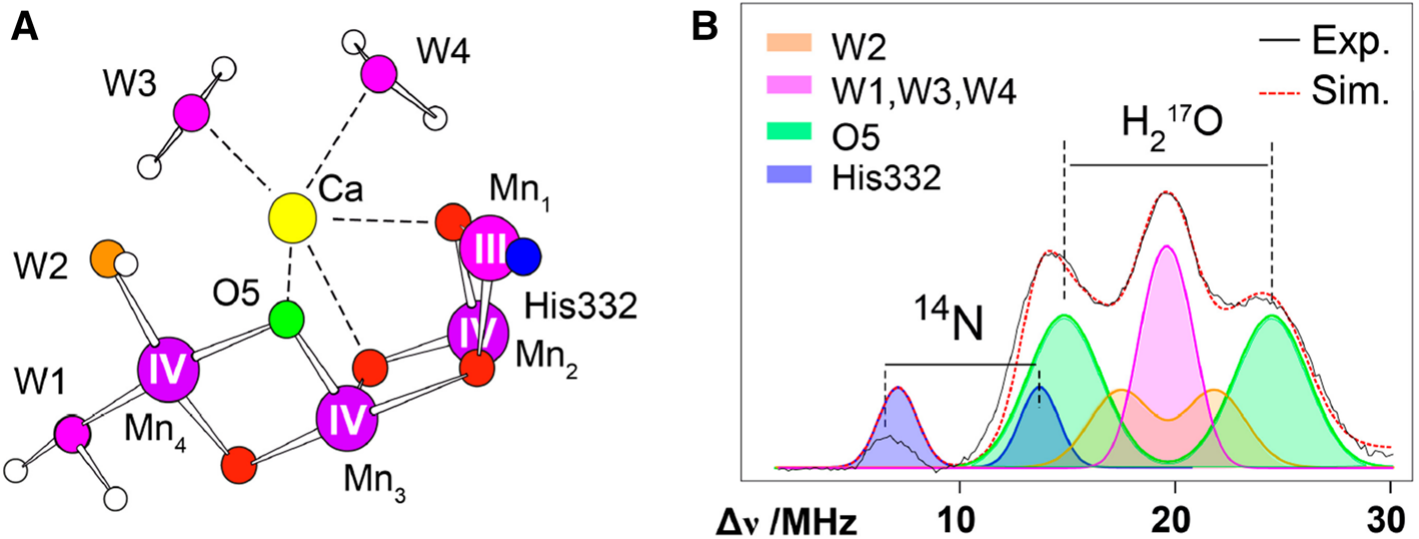
**A** Mn cluster with the µ-oxo bridges and bound H_2_O/OH^−^ molecules in the S_2_ state; the same color code as in panel (**B**) is used. **B**^17^O EDNMR spectrum at W-band (94 GHz) obtained from PS II core preparations (*T. elongatus*) in the S_2_ state. The buffer has been exchanged with H_2_^17^O (90% enrichment) for 30 min (3 ×) in the dark (*S*_1_ state) prior to flash-advancement to the *S*_2_ state (black trace). EDNMR spectra are also shown for H_2_^16^O buffer, containing only ^14^N signals (dark blue trace, ^14^N from histidine His332, see Fig. 2C). Three classes of oxygen ligands were assigned: the largest ^17^O splitting belongs to the exchangeable µ-oxo bridge O5 (green), the smaller ones to the H_2_O/OH^−^ molecules (orange, purple) indicated in the figure. For further details see (Rapatskiy et al. 2012)

For the detection of substrate water molecules, EPR spectroscopy makes use of interactions with nuclear spins, e.g. ^2^H (in ^2^H_2_O) (Fiege et al. 1996; Kawamori et al. 1989; Nagashima and Mino 2013) or preferentially ^17^O (nuclear spin *I* = 5/2) in PS II samples with isotope-labeled H_2_^17^O as the solvent (Lohmiller et al. 2017; McConnell et al. 2012; Rapatskiy et al. 2012). Methodological and instrumental developments in our laboratory (Cox et al. 2013a, 2017) allowed us to characterize the ^17^O signals in the S_2_ and S_0_ states using electron–electron double resonance (ELDOR)-detected NMR (EDNMR) at high frequency (94 GHz) (Lohmiller et al. 2017; Rapatskiy et al. 2012), which yields significantly better sensitivity compared with ^17^O ENDOR (McConnell et al. 2012; Rapatskiy et al. 2012). Through spectral simulations, we could identify the µ-oxo bridge O5 to be an exchangeable ligand in the *S*_2_ state (Fig. 7A, B) (Lohmiller et al. 2014; Perez-Navarro et al. 2013; Rapatskiy et al. 2012). O5 represents the most probable candidate for the two-fold deprotonated substrate W_s_ for the following reasons:

(i) It is the only exchangeable ^17^O nucleus bound both to Mn and Ca, a requirement imposed by MIMS experiments (Cox and Messinger 2013; Hendry and Wydrzynski 2003; Messinger 2004).
(ii) Shortening of an Mn–Mn bond observed by EXAFS has been explained by a deprotonation of an O-bridge and has been assigned to O5 (Robblee et al. 2002; Messinger 2004).
(iii) Similar timescales for the water exchange were found both for W_s_ in the MIMS, see ref. (Hillier and Wydrzynski 2008), and for O5 in the EPR experiments, i.e., complete exchange in < 10 s (Rapatskiy et al. 2012).
(iv) Orientation dependent EDNMR studies showed that the measured hyperfine coupling can only be assigned to O5 or O4) (Rapatskiy et al. 2012).
(v) Both Ca/Sr exchange experiments and ammonia inhibition studies demonstrate O5 and only O5 to be an exchangeable oxygen bridge (Cox et al. 2011; Perez-Navarro et al. 2013).

This is in agreement with earlier low frequency FTIR studies of the OEC that showed that a µ_2_-oxo or µ_3_-oxo bridge is exchangeable (Chu et al. 2000). It is remarkable that in the OEC a µ-oxo bridge is readily exchangeable. For structurally similar model compounds an exchange has only been observed on much longer timescales (Rapatskiy et al. 2015; Tagore et al. 2006, 2007). These results further demonstrate the dynamic nature of this O5 ligand in the OEC (see above, Fig. 5).

**Fig. 8:**
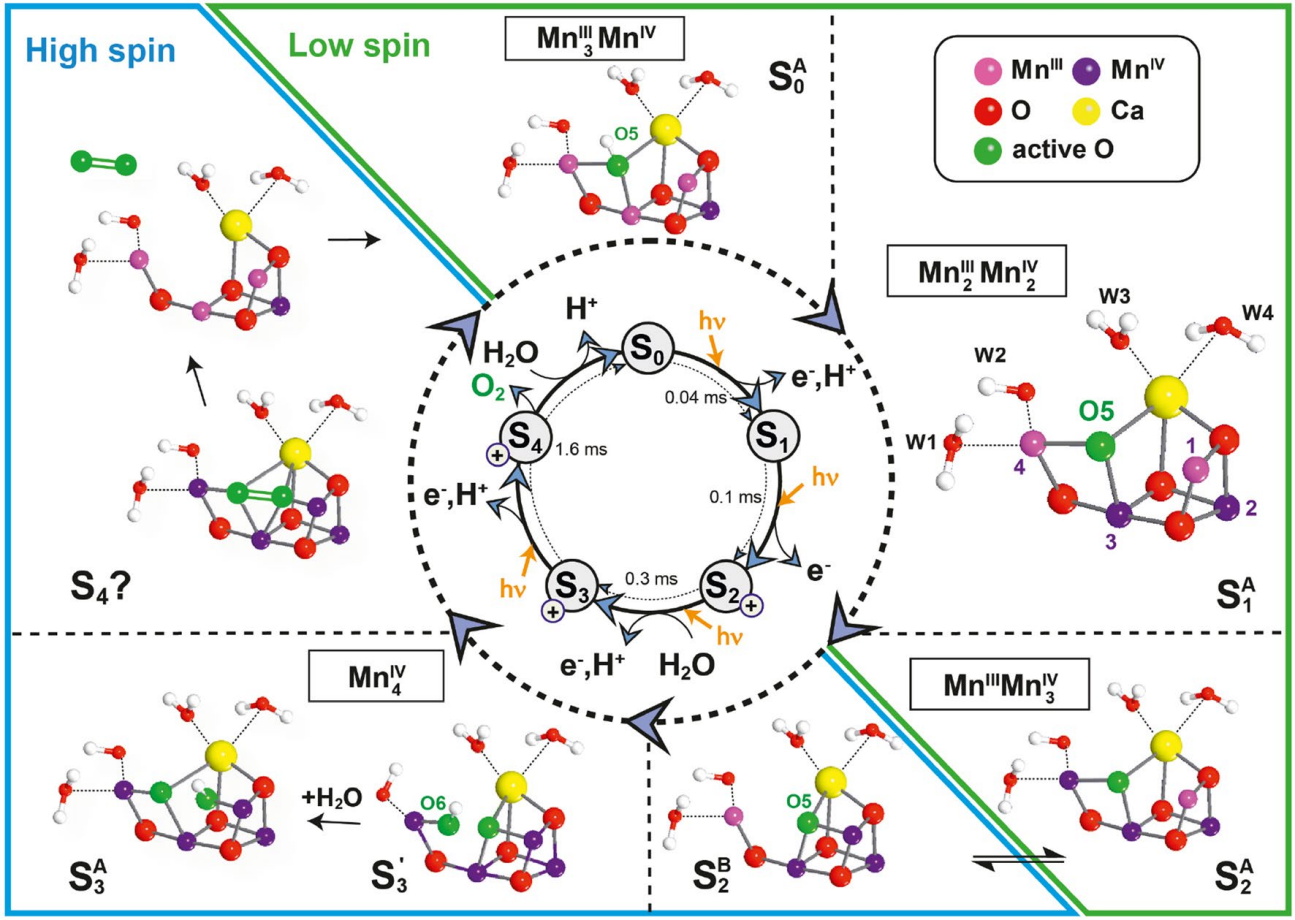
Model for the water oxidation cycle in PS II based on spectroscopic and theoretical work (Cox et al. 2015; Krewald et al. 2016) detailing the structures of the Mn cluster in the different *S* states, the water binding events and the O–O bond formation and O_2_ release in *S*_4_. The boxes show the determined oxidation states of the Mn ions in the respective *S* state. A color code is used for the assignment of the Mn^III^ (light purple) and Mn^IV^ (dark purple) ions when the OEC is passing through the *S* states. Note that the oxygens of the waters and µ-oxo bridges are given in red except for the proposed substrate oxygens O5 and O6 (green). The *S*_2_ state exists in two conformations (closed/open cubanes), see text. *S*_3_ may also exist in an open (*S*_3_^A^) and a closed (*S*_3_^B^) cubane form (not shown). For *S*_3_ a state *S*_3_′ is shown in which manganese oxidation has occurred but the 2nd water has not been introduced. This state is present in native sample in a minority of PS II centers, but it is stabilized upon specific chemical treatment (methanol addition or Ca^2+^ to Sr^2+^ exchange) (Chrysina et al. 2019). A switching of the preferred total spin ground state configuration of the cluster is thought to take place in *S*_2_ from low to high spin and between S_4_ and S_0_ back from high to low spin; this is indicated by the diagonal line dividing the green and blue boxes. This switching of the spin state may be necessary for the formation of triplet dioxygen ^3^O_2_ in the final step of the cycle (see also Fig. 10). Figure changed from (Krewald et al. 2016)

Recent ^17^O EDNMR investigations of H_2_^17^O binding to the *S*_0_ state of the OEC show that an OH^−^ is introduced in the initial step of the cycle (the 1st substrate water that has lost a proton) after O_2_ release in the *S*_4_ to *S*_0_ transition (Lohmiller et al. 2017). In the *S*_0_ and *S*_1_ state the O5 is thought to exist in an open cubane-type structure (in *S*_0_ with *S* = ½, and in *S*_1_ with *S* = 0 ground state) as shown in Fig. 8.

As described above, recent spectroscopic results, including high field EPR indicate that a new water molecule (O6) is inserted into the manganese cluster in the *S*_3_ state as an OH^−^ at the open coordination site of Mn1 (Cox et al. 2014). Its close proximity to O5 suggests it is the second substrate *W*_f_ (Fig. 6B). How it is exactly inserted remains an open question. Recent SFX data (Kern et al. 2018; Suga et al. 2017) indicate that the Glu189, which bridges the Ca^2+^ ion and Mn1 in the *S*_1_ state detaches from the Ca^2+^ ion upon formation of the *S*_3_ state. This removes steric hindrance to direct water binding via the Ca^2+^ channel, i.e., it is originally one of the Ca^2+^ waters. Another possibility is that this OH^−^ (O6) is introduced in an indirect fashion by toggling between the open and closed cubane structures and is thus derived from W2, one of the terminal ligands of Mn4 (compare structure *S*_3_′ in Fig. 8). Within this sequence the water that binds during the *S*_2_ to *S*_3_ transition is not one of the substrates of the current cycle, but a substrate of the next cycle (Cox and Messinger 2013). It also implies that O5 (the existing μ-oxo bridge) and O6 (the new water-derived oxygen ligand) may switch identities. There is significant theoretical support for this sequence, which explains how redox tuning of the cofactor can precede water binding (Retegan et al. 2016). This hypothesis also better agrees with MIMS data which shows both substrates are bound to the cofactor (most likely to Mn ions) in all *S*-states and that the rate of exchange does not dramatically change upon moving from *S*_2_ to *S*_3_ (Cox and Messinger 2013; Hendry and Wydrzynski 2002, 2004).

## The water oxidation cycle in PS II

As a consequence of the above results, it is assumed in the following discussion of the catalytic cycle of the OEC that the O5 bridge derives from one of the substrate waters (W_s_). A possible cycle is shown in Fig. 8 (Cox et al. 2015; Krewald et al. 2016). The first water W_s_ binds as an OH^−^ (O5) in the *S*_0_ state, being deprotonated during the subsequent oxidation step to *S*_1_. In the *S*_3_ state the second substrate water enters the reaction as OH^−^ (O6) to Mn4 (or Mn1) as suggested by the results obtained from EPR and XFEL experiments on the S_3_ state (Cox et al. 2014; Kern et al. 2018; Suga et al. 2017). A further oxidation and deprotonation step leads to *S*_4_. Experimental evidence shows that formation of *S*_4_ is triggered by proton release (Haumann et al. 2005a; Klauss et al. 2012a). Current theoretical models suggest that a proton is transferred from W1 (H_2_O) to Asp61, as in the preceding *S*_2_ to *S*_3_ transition (Siegbahn 2012). Mutants of PSII lacking Asp61 show a pH dependency of the lag phase during the *S*_3_–*S*_0_ transition which has been attributed to a deprotonation of the Mn_4_CaO_5_ (Bao and Burnap 2015; Dilbeck et al. 2012) in support of this model.

In the current literature, there are several proposed mechanisms that suggest the early onset of O–O bond formation in the S_3_ state (Corry and O’Malley 2018). This is mainly based on the SFX crystal structure of Suga et al. which modeled a short distance between the O5 and O6 bridges in the *S*_3_ state (Suga et al. 2017). We note that the more recent higher resolution SFX structure of the *S*_3_ state (Kern et al. 2018), does not exhibit this short O5–O6 distance, and is instead more consistent with the high field EPR structure described above. Furthermore, earlier biophysical measurements categorically rule out O–O bond formation in the *S*_3_ state. The MIMS data described above shows that both substrates still rapidly exchange with bulk water in the *S*_3_ state (Messinger et al. 1995). It is only upon oxidation and proton release to form the *S*_3_*Y*_z_^·^ state directly preceding *S*_4_ that substrate exchange is ‘arrested’ indicating the onset of O–O bond formation, i.e., formation of a peroxo type intermediate (Nilsson et al. 2014).

How the O–O bond is precisely formed in the *S*_4_ state is still unknown, owing to a lack of experimental data. There are two popular chemical mechanisms for this transition that differ with regard to what component of the cofactor is oxidized to form *S*_4_: (i) a ligand (one of the substrate waters); or (ii) a metal (one of the Mn ions). In the case of a ligand oxidation event, forming a Mn^IV^-oxyl moiety, O–O bond formation is proposed to proceed by a radical coupling mechanism described below. In the case of a metal oxidation event, forming a Mn^V^-oxo type moiety, O–O bond formation is instead proposed to proceed by nucleophilic attack of the electrophilic oxygen (O5) bound to the Mn^V^ by a nearby water, e.g., W3 bound to the Ca^2+^. Unfortunately the nature of the last oxidation (ligand vs. metal centered) does not resolve this question. XAS data which monitors an oxidation state change of the Mn ions during the *S*_3_ to *S*_0_ transition appears to rule out a Mn^V^ intermediate (Haumann et al. 2005a), favoring instead a ligand (oxygen) oxidation. We note that while the nucleophilic attack mechanism initially envisaged the involvement of a Mn^V^ intermediate, it was later suggested to have substantial Mn^IV^ oxyl character in the *S*_4_ state based on theoretical studies (Vinyard and Brudvig 2017).

Only a nucleophilic attack mechanism has been observed for first row molecular transition metal water oxidation catalysts, for which there is mechanistic data, (Codola et al. 2015) with radical coupling to be thus far only described for second row transition metals, e.g., ruthenium (Cox and Lubitz 2013; Romain et al. 2009). Nucleophilic attack mechanisms have thus been proposed for the OEC by a number of groups (Ferreira et al. 2004; Pecoraro et al. 1998); see (Barber 2017; Vinyard and Brudvig 2017; Vinyard et al. 2015) for recent reviews. In this sequence, a nearby water (e.g., W3) attacks a manganese bound oxygen, most likely O5. Upon re-reduction of the four Mn ion and loss of the product O_2_, the newly inserted O6 bridge presumably fills the site vacated by O5 leading to rapid recovery of the *S*_0_ state.

In contrast the oxo-oxyl radical coupling mechanism involving two Mn bound oxygens, is most compatible with the assignment of the two substrates sites described in the previous section (O5 and O6). Such a mechanism was first proposed ten years ago by Per Siegbahn (Siegbahn 2009). Importantly high field *S*_3_ state EPR data has demonstrated that there is the requisite spin alignment of the two putative substrate oxygens and the Mn ions to which they are bound (Mn4-O5-O6-Mn1 = βαβα) on which this mechanism is based.

**Fig. 9:**
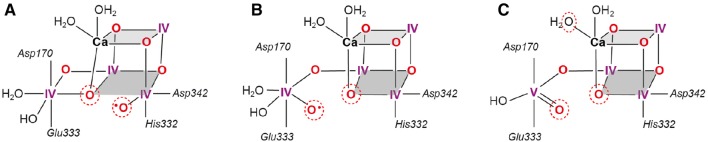
Three representative model structures for the final step in the Kok cycle with O5 and O6 bound (see Figs. 8, 10); **A** Mn(IV)-oxyl formation at Mn1, octahedral coordination, all Mn(IV), open cubane structure; **B** Mn(IV)-oxyl formation at Mn4, octahedral, closed cubane structure; **C** Mn(V) = O formation at Mn4, trigonal bipyramidal, closed cubane. The putative reacting oxygens are indicated by dotted red circles; for details see text. Figure adopted from (Pantazis 2018)

To illustrate the current situation, in Fig. 9 three different structures are shown preceding the O–O bond formation in *S*_4_. They are derived from the *S*_3_ open and closed cubane structures, *S*_3_^A^ and *S*_3_^B^, and from *S*_3_′ in which a second water has not (yet) been bound (Fig. 8). In panel (A) and (B) the open and closed cubane structures are shown with all Mn(IV) and O5 and O6 in close contact that could react to form dioxygen via an oxyl-oxo coupling mechanism. In panel C) the dangling Mn4 is 5-coordinate; in the shown geometry the formation of a Mn(V) = O species is preferred in which the electrophilic oxygen could be attacked by a nucleophilic water (e.g., bound to Ca) or a neighboring oxo group as pointed out by Pantazis (2018). In this context it should be mentioned that in the recent SFX structure (Kern et al. 2018) only the open cubane structure has been observed for *S*_2_ and *S*_3_ which led the authors to propose O5 to react with either O6 (here called Ox) bound to Mn1 or with W3 (at Ca) or W2 (at Mn4).

There are also other alternative mechanisms. A very recent example, proposed by the Yamaguchi group, involves an O–O˙ radical intermediate, formed following electron transfer to *Y*_Z_ (Shoji et al. 2018). Similarly, there are proposed mechanisms that do not fall into either of the two categories, such as a recent proposal from the Sun group. In this novel mechanism, the dangler Mn acts as the site of catalysis, as in a nucleophilic attack like mechanism, forming a Mn^VII^-dioxo intermediate following charge rearrangement of the Mn cluster in the *S*_4_ state (Zhang and Sun 2018). As above for the nucleophilic attack mechanism, it is unclear if this mechanism is compatible with XAS data (Haumann et al. 2005a). For a more detailed comparison of alternative mechanisms the reader is directed to a recent in-depth review (Pantazis 2018).

**Fig. 10:**
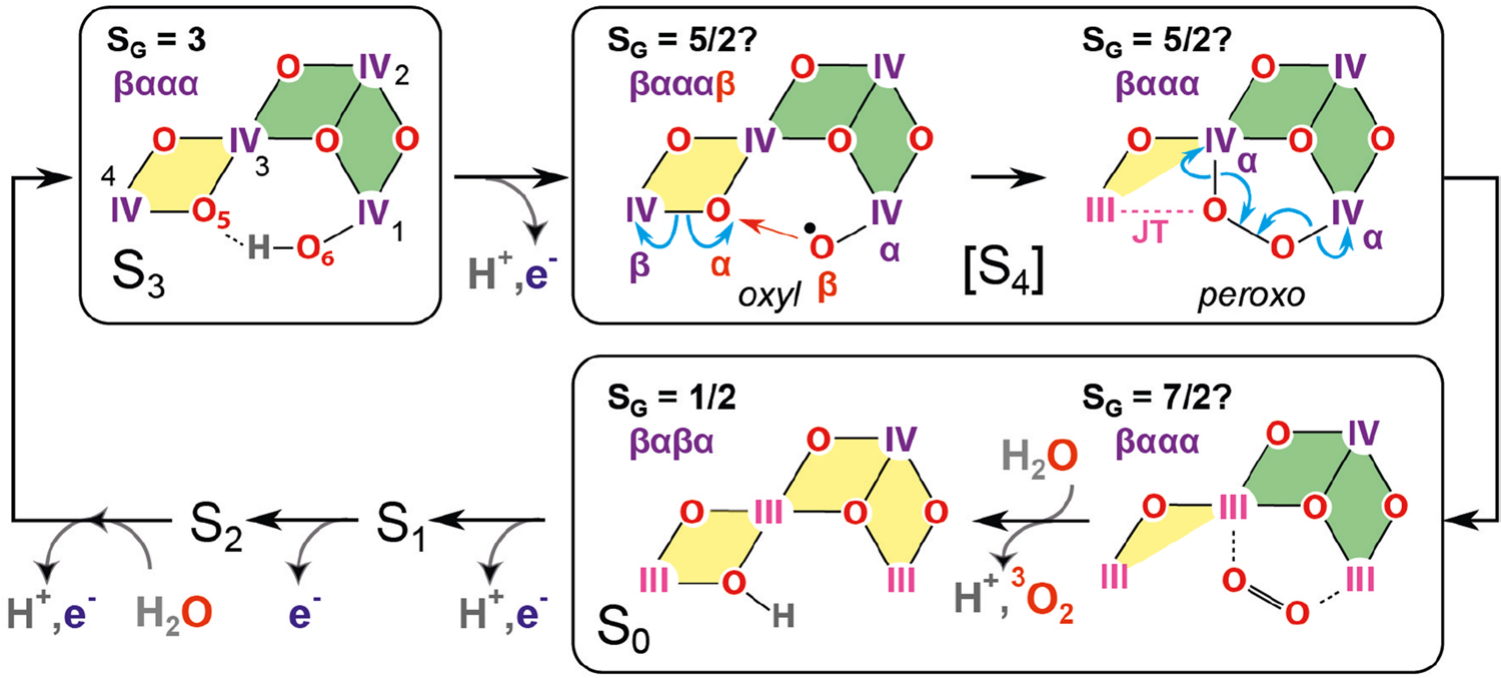
Spin alignment in the *S*_3_, [*S*_4_] and *S*_0_ states that fosters low barrier O–O bond formation as described by Siegbahn (Siegbahn 2009). *S*_3_ and *S*_0_ have been characterized experimentally; [*S*_4_] has not been observed. The spin ground state *S*_G_ is indicated. The numbering of the Mn ions (1–4) is given for the *S*_3_ state; the oxidation state of each Mn ion is given in light purple (III) or dark purple (IV); antiferromagnetic interaction between adjacent Mn ions is represented by yellow shading whereas ferromagnetic interaction by green shading. The spin alignment of the Mn ions in each state is given on the top (the order is: Mn4-Mn3-Mn2-Mn1); the spin of the oxyl radical at the [*S*_4_ oxyl] state is indicated by the red colored β. The Jahn–Teller (JT) axis of the Mn^III^ of the [*S*_4_ peroxo] intermediate is depicted by a dashed purple line. Evidence of the spin alignment of Mn4-O5-O6-Mn1 in the [*S*_4_ oxyl] state (βαβα) is derived from experiments on the *S*_3_ state (Cox et al. 2014; Krewald et al. 2016). For details of the mechanism see text

Owing to the increasing experimental support for the oxo-oxyl coupling mechanism first proposed by Siegbahn, a brief description of its key steps is given below (Fig. 10). The first stage of the mechanism involves oxidation of O6 to form an oxyl radical, in concert with deprotonation. In the Siegbahn mechanism, the proton associated with O6 in *S*_3_ migrates to W1, which is already deprotonated during *S*_3_ formation. The generated oxyl radical carries an unpaired β spin electron, because O6 is antiferromagnetically coupled to Mn1. The oxyl radical then attacks the O5 bridge which carries a small excess of α spin. This results in formation of an σ O–O bond between O5 and O6—with the new σ bond consisting of the α spin electron of O5 and the β spin electron of O_6_ (Fig. 10 [*S*_4_ oxyl]). The α spin electron comes from the cleavage of the existing Mn–O bond between O5 and Mn4. The β spin electron of the cleaved Mn–O bond is transferred back to Mn4, reducing its oxidation state to III+. Note that the β electron of the cleaved Mn–O bond has the same spin as the three existing d-electrons of Mn4, and thus this transfer can occur without energy penalty due to spin conversion.

Subsequently, a peroxo product (a bound O_2_ with a single σ bond) is formed bridging Mn3 and Mn1 (Fig. 10 [S_4_ peroxo]). Mn4 is now five coordinate with the product peroxide sitting along its Jahn–Teller (JT) axis (Li and Siegbahn 2015; Siegbahn 2009). Cleavage of the two remaining Mn–O bonds leads to formation of the final O_2_ product. Importantly the cleavage of each Mn–O bond results in an α spin electron being transferred to each of the two Mn ions that were attached to the peroxide and a β spin electron being transferred to O5 and O6. As before, this is an energetically favorable situation as the electron gained by each metal is of the same spin (α) as their existing *d*-electrons. The two new β spin electrons occupy two π* orbitals giving rise to the triplet O_2_ product. The displacement of O_2_, via binding of the first substrate water of the next S cycle (coupled to its deprotonation), leads to formation of the observed (metastable) *S*_0_ state (Fig. 10, *S*_0_). The O_2_ may detach in a stepwise fashion, during its concerted replacement with OH^−^, passing through a transient superoxo intermediate (Li and Siegbahn 2015).

New experimental data is needed to probe these key stages in O–O bond formation and release. SFX crystallography (Kern et al. 2014, 2018; Kupitz et al. 2014; Suga et al. 2015, 2017; Young et al. 2016) is poised to address structural changes during the *S*_3_ to *S*_0_ transition, and may suggest an alternative proton transfer sequence. Specifically, as described above, the Siegbahn mechanism starts with internal proton transfer from O6 to W1. We note, however, that recent SFX measurements indicate that Glu189 detaches from the Ca^2+^ ion during S-state progression. Glu189 is proximal to Mn1 and could act as an internal base, accepting a proton from O_6_, similar to old ideas put forward by Gerry Babcock (Hoganson and Babcock 1997). Within this revised scheme W1 does not need to undergo deprotonation—with proton transfer to Asp61 switched off due to a conformational change (gating) (Kern et al. 2018).

## Conclusions

In biology, there is only one catalytic site that is able to split water—the OEC—and its structure and function is identical (Su et al. 2011) in cyanobacteria, algae and all higher plants; however, differences in the second coordination sphere between higher plants and cyanobacteria have been reported (Retegan and Pantazis 2017). Many other enzymes have found different ways to perform their task—but not the unique, highly optimized OEC in PS II. The work compiled above shows that this unique metal cluster requires the presence and concerted action of all four manganese ions in the Mn_4_O_x_Ca complex. The oxidation states of the Mn ions are always III or IV—there exists no Mn^II^ in the whole cycle. The possible presence of a Mn^V^ in the last step (S_4_) has, however, not yet been finally clarified. The importance of the total electron spin state of the cluster has been highlighted—and is understandable considering the necessary restriction to form and release triplet dioxygen ^3^O_2_. The Ca^2+^ is probably important for efficient water delivery and the Cl^−^ for maintaining the correct charge balance of the OEC. Water delivery is optimized through highly efficient water channels leading to the Mn cluster. In the favored model the two substrate waters bind to neighboring redox-active Mn ions, and deprotonation of these waters is coupled to the oxidation events of the cluster. Close proximity and proper alignment of the two active oxygens are assured to efficiently form the O–O bond and finally release the ^3^O_2_ molecule with high efficiency.

In spite of the great progress in the understanding of the OEC in PS II there are still several challenges to be tackled. One pertinent open question is the structure of the elusive S_4_ state. A possible stabilization and characterization of this state would finally complete our knowledge of the S states of the Kok cycle. A missing key experiment is further the detection of the 6th oxygen in the manganese cluster by EPR (using ^17^O labeling) to support the recent crystallographic XFEL (SFX) data on the *S*_3_ state (Kern et al. 2018; Suga et al. 2017). Furthermore the determination of the water binding kinetics should be followed for all state transitions by rapid freeze quench (RFQ) techniques combined with advanced EPR techniques. First experiments along these lines have successfully been performed in our laboratory using time resolved RFQ ^17^O EDNMR measurements (Rapatskiy et al., unpublished data). The exchange of certain amino acids in the vicinity of the OEC could shed light on the fine-tuning of its electronic structure in the S state cycle and would be important for explaining its high turnover frequency. Furthermore, a great challenge for chemists working in the field of water oxidation is the understanding and modeling of the sophisticated mechanism by which PS II repairs photo-damage of its protein, of the pigments and of the manganese cluster.

The properties of the OEC in PS II described above represent valuable design features for bioinspired light-driven molecular catalysts for water oxidation. The following points are considered essential for such catalysts:

1. The material of the catalyst must be abundant, easily accessible, inexpensive (i.e., no precious metals), non-toxic, sufficiently stable under working conditions and should be scalable.
2. The required four oxidizing equivalents must be stored in the catalyst to couple the fast 1-electron photochemical reaction with the slow 4-electron chemical water oxidation process.
3. The binding of the two substrate water molecules should preferentially take place at two well-defined neighboring redox-active metal centers, accompanied by successive deprotonation and activation of the water molecules.
4. The redox steps of the catalytic metal centers should be of similar magnitude and in the range of 1 eV, and should finally lead to a concerted water oxidation, O_2_ formation and O_2_ release, to avoid reactive oxygen intermediates. This also requires a sequential release of the 4 protons.
5. A functional matrix mimicking the protein (in PS II) is needed for efficient transport of water to the reaction zone, release of dioxygen and for the correct proton management.
6. In the photochemical act, a light-induced species must be generated with sufficient oxidative power to oxidize water (+ 1.23 eV).
7. Effective coupling of the charge separation unit with the catalytic center (metal cluster) is necessary to diminish the overpotential (comparable to the function of the tyrosine *Y*_Z_ in PS II).
8. The catalytic unit should be stable; in case of deterioration/destruction a mechanism of self-repair or healing should be in place to avoid deactivation of the catalytic process.

At present, it is still a great challenge to create a catalyst that fulfills the above criteria and could thus compete with the Mn cluster in PS II. The future will show if at least part of the above points can be fulfilled. Attempts to synthesize water splitting chemical catalysts and devices are too numerous in the literature to be discussed here—but show the great importance of this research field for a future society using (only) renewable, sustainable energy based on sunlight driven processes (Andreiadis et al. 2011; Blakemore et al. 2015; Kanan and Nocera 2008; Kurz 2016; Najafpour et al. 2016; Tran et al. 2012; Zhang et al. 2015; Matheu et al. 2019).
